# ADHD-related mental health and school impairments across educational stages: sex-specific patterns in Japanese youth

**DOI:** 10.3389/fpsyt.2026.1736727

**Published:** 2026-10-22

**Authors:** Fumito Takahashi

**Affiliations:** Institute of Education, Shinshu University, Nagano, Japan

**Keywords:** academic, anxiety, attention-deficit/hyperactivity disorder, bullying victimization, depression, friend, irritability, teacher

## Abstract

**Introduction:**

While ADHD-related challenges are well-documented internationally, understanding of how these manifest across educational stages and sexes remains limited. This study examined mental health symptoms and school functioning in Japanese children with and without ADHD across elementary, junior high, and senior high school.

**Methods:**

In this comparative cross-sectional study, parents of 1,594 Japanese children (797 with ADHD, 797 without ADHD) aged 6–18 years completed questionnaires assessing ADHD symptoms (ADHD-RS-IV), depression (SMFQ), anxiety (SCAS), irritability (ARI), bullying victimization, academic achievement, and social support. Three-way ANOVAs examined interactions between ADHD status, child sex, and educational stage, with Cohen's d effect sizes calculated for group comparisons.

**Results:**

Children with ADHD demonstrated significantly poorer outcomes across nearly all domains, except for teacher support, with effect sizes ranging from moderate to very large (d = 0.35–1.44). Contrary to expectations, the largest group difference for girls' depression emerged in elementary school (d = 1.44) rather than junior high school, and girls' anxiety differences were comparably large in elementary (d = 1.25) and junior high school (d = 1.30). Girls with ADHD showed consistently larger effect sizes than boys for depression (mean d: 1.14 vs. 0.84), anxiety (1.13 vs. 0.73), and irritability (1.32 vs. 0.90) across all stages. Significant ADHD × sex interactions emerged for depression, anxiety, and irritability. Bullying victimization affected 31.0% of children with ADHD compared to 7.5% of non-ADHD children, with peak effects in elementary school. Academic difficulties showed the anticipated pattern of increasing impairment from elementary to junior high school, particularly for boys (d = 0.65 to 1.07).

**Discussion:**

These findings reveal pronounced sex differences in ADHD-related mental health burden, with girls experiencing what may be a "hidden burden" of greater internalizing symptoms across development. The smaller group differences for internalizing symptoms in adolescence may partly reflect decreased parental awareness of adolescent internalizing symptoms. These results underscore the need for sex-specific, developmentally-informed interventions with particular attention to early identification and support for girls with ADHD who may be overlooked in clinical and educational settings.

## 1 Introduction

Different educational stages—elementary, junior high, and senior high school—represent distinct developmental contexts, each characterized by unique academic, social, and emotional challenges associated with varying levels of mental health difficulties (1, 2). Research demonstrates that approximately 46% of young people exhibit elevated mental health symptoms during secondary school, with different educational stages showing distinct patterns of vulnerability and resilience (3). For children with Attention-Deficit/Hyperactivity Disorder (ADHD), these contexts present varying levels of challenge and support that differentially affect their vulnerability to mental health problems and social difficulties. The environmental characteristics of each stage—from single-teacher elementary structures to multiple-teacher secondary environments—are associated with different levels of symptom expression and impairment (4).

ADHD affects approximately 5-7% of school-aged children globally (5, 6), with recent umbrella reviews confirming 8.0% prevalence across 588 primary studies (7). Similar prevalence rates are reported in Japan, with municipal studies documenting 5.8% prevalence (8) and 6.27% of Japanese university students meeting childhood ADHD criteria (9). Beyond core symptoms of inattention, hyperactivity, and impulsivity, children with ADHD experience significant emotional challenges profoundly impacting their wellbeing.

Among these emotional challenges, depression represents one of the most significant mental health comorbidities, affecting 14% of children with ADHD versus substantially lower rates in general populations (10), with prevalence increasing to 9-50% in adolescents (11). Japanese studies reveal even more pronounced patterns, with adults with ADHD showing 60-fold higher depression rates compared to controls (12). Anxiety disorders show similarly elevated prevalence, affecting 18% of children with ADHD versus 2% without (10), increasing to 27.4% in early adolescents (13) and reaching 47% in adults (14). Japanese studies demonstrate 40-fold higher anxiety rates in adults with ADHD (12). Additionally, irritability represents a key dimension of emotional dysregulation, with 91% of children with ADHD endorsing at least one irritable symptom and 31% meeting criteria for Disruptive Mood Dysregulation Disorder (15). Research established that severe mood dysregulation youth have high rates (27%) of ADHD (16), while meta-analysis demonstrate emotion dysregulation affects 24-50% of children with ADHD, representing a major contributor to functional impairment (17).

These emotional challenges are compounded by significant social and academic difficulties. Children with ADHD face dramatically increased bullying victimization, being 2.9 times more likely to experience “sometimes” bullying and 10.8 times more likely to experience “often” bullying compared to peers (18). Recent data shows 46.9% of children with ADHD experiencing bullying versus 23% in general populations (19), with clinical studies revealing 14.6% pure victims, 8.4% pure perpetrators, and 5.6% victim-perpetrators among adolescents with ADHD (20).

In addition to heightened victimization risk, children with ADHD face substantial academic challenges, demonstrating performance 0.71 standard deviations below average (21), with systematic reviews of 176 studies confirming achievement test outcomes are 79% worse and academic performance 75% worse in untreated ADHD (22). Meta-analysis of 83 studies encompassing 3,734 children demonstrated significant impairment across all executive function domains, with effect sizes ranging from 0.46-0.69 standard deviations (23). By high school, students with ADHD show significantly lower GPAs and are 8.1 times more likely to drop out (14.1% vs 1.4%) (24).

Social challenges extend beyond victimization to fundamental relationship difficulties. The Multimodal Treatment Study demonstrates 56% of children with ADHD have no reciprocated friendships versus 32% of typically developing peers (25), with reviews finding up to 76% of children with ADHD lack reciprocated friendships (26). Peer rejection is associated with cigarette smoking, delinquency, anxiety, and global impairment in adolescents with ADHD (27). Teacher relationships are characterized by low closeness (r = -0.170) and high conflict (r = 0.414) according to meta-analysis of 27 studies with over 17,000 participants (28), with reduced teacher support correlating with greater behavioral difficulties (29).

Girls with ADHD face unique challenges often underrecognized clinically. Hinshaw et al. (30) demonstrated girls show predominance of inattention and internalizing problems, with compensatory strategies masking impairments despite significant functional difficulties. Meta-analysis found girls with ADHD were 3.2 times more likely to have anxiety disorders and 4.2 times more likely to have depression compared to non-ADHD girls (31). Irritability manifests differently by sex, with twin genetic studies revealing girls show stronger irritable mood than boys (32), and the association between irritability and internalizing symptoms being stronger in girls (33). Longitudinal examination revealed irritability was associated with higher relative hyperactive/impulsive symptoms specifically in females (34). Girls with ADHD also experience significantly higher rates of both relational and overt victimization (35), with pathways from ADHD symptoms through peer dislike and victimization to depression present specifically in girls but not boys (36).

The emotional, social, and academic challenges described above do not remain static but evolve as children with ADHD navigate successive educational environments, each imposing distinct demands. In elementary school, single-classroom-teacher structures offer relatively close monitoring and consistent routines; nevertheless, longitudinal research following over 2,000 children demonstrated that high inattention levels during this period are already associated with significantly lower educational attainment in adulthood (37), indicating that the protective features of elementary settings do not fully buffer against ADHD-related risk. The transition to junior high school introduces multiple subject-specific teachers, reduced individual oversight, and substantially greater organizational demands—conditions that the Multimodal Treatment Study identified as particularly challenging for students with ADHD, who show distinct patterns of symptom exacerbation in these settings (4). Senior high school further compounds these pressures through intensified academic competition and greater expectations for self-regulation, while simultaneously granting adolescents more autonomy that may both benefit and challenge those with ADHD. Together, these stage-specific environmental shifts suggest that the magnitude and nature of ADHD-related difficulties may vary systematically across the educational trajectory, yet few studies have examined these patterns simultaneously within a single sample.

The need for such comprehensive examination is further underscored by cultural context, which significantly influences ADHD recognition and management. Despite similar symptom prevalence, ADHD medication use in Japanese children is only 0.4% compared to 5.3% in the US, suggesting substantial underdiagnosis and undertreatment (38). Recent nationwide studies found autism spectrum disorder (ASD) as the most common comorbidity (54.4%) in Japanese children with ADHD, potentially reflecting diagnostic practices where ADHD is underdiagnosed relative to ASD (39). Given these cultural factors, dramatic elevations in comorbidity rates when ADHD is identified suggest Japanese girls and women with diagnosed ADHD may represent particularly severe cases.

Given that Japanese children with ADHD face well-documented multi-domain impairments within a cultural context of substantial underdiagnosis and undertreatment, systematic examination of how these challenges manifest across educational stages and sexes is needed. This cross-sectional study aims to comprehensively compare school experiences and mental health outcomes of Japanese children with and without ADHD across elementary, junior high, and senior high school periods, with particular attention to sex-specific patterns. Drawing on the consistent evidence of elevated emotional comorbidities and functional impairments reviewed above (e.g., 10, 17, 22), we hypothesize that (1) children with ADHD will demonstrate significantly poorer outcomes across all measured domains. Based on evidence that successive educational transitions impose increasing organizational and self-regulatory demands on students with ADHD (4, 37), we further hypothesize that (2) the magnitude of ADHD–non-ADHD group differences will vary by educational stage, with junior high school showing the most pronounced differences given its increased environmental demands. Finally, consistent with meta-analytic findings of heightened internalizing comorbidities in girls with ADHD (30, 31), we hypothesize that (3) psychological distress associated with ADHD will be particularly pronounced in girls. Understanding these stage-specific and sex-specific patterns is crucial for identifying optimal timing and targets for intervention and informing educational policies supporting students with ADHD throughout their academic careers.

## 2 Materials and methods

### 2.1 Study design and setting

This comparative cross-sectional study, in which participants were recruited according to parent-reported ADHD diagnosis status, was conducted in Japan during March 2021 through an online survey platform. The study aimed to examine mental health symptoms and associated factors among Japanese school-aged children with and without ADHD diagnosis from elementary through high school, capturing a critical developmental period marked by multiple educational transitions. The Japanese educational system comprises six years of elementary school (grades 1-6, ages 6-12), three years of junior high school (grades 7-9, ages 12-15), and three years of senior high school (grades 10-12, ages 15-18), with major transitions occurring between elementary and junior high school, and between junior and senior high school.

### 2.2 Participants

#### 2.2.1 Sample size and recruitment

A total of 1,600 parents of school-aged children participated in this cross-sectional online survey, comprising two distinct groups. The ADHD group consisted of 800 parents who reported having at least one child with a formal ADHD diagnosis. In cases where multiple children had ADHD diagnoses, parents were asked to provide information about their oldest child with ADHD to ensure consistency in selection criteria. The non-ADHD (control) group comprised 800 parents who reported having no children with an ADHD diagnosis. These parents were asked to provide information about their oldest school-aged child to ensure consistency and avoid confusion in response selection.

Participants were recruited through Rakuten Insight Inc., one of Japan’s largest online research panel companies with over 2.2 million registered panelists, ensuring demographic diversity. The recruitment process involved a two-stage screening. In the initial screening stage, parents of school-aged children were asked whether any of their children had a formal diagnosis of ADHD. Based on their response, they were allocated to either the ADHD group or the potential non-ADHD group. In the group assignment stage, the first 800 eligible parents who answered “yes” were enrolled in the ADHD group and asked to complete the survey regarding their oldest child with ADHD. The first 800 eligible parents who answered “no” were enrolled in the non-ADHD group and asked to complete the survey regarding their oldest school-aged child without ADHD.

The inclusion criteria were: (1) being a parent or primary caregiver of at least one child currently enrolled in grades 1–12 in the Japanese education system, (2) for the ADHD group, having a child with a formal ADHD diagnosis from a qualified healthcare professional, (3) residing in Japan, (4) ability to read and respond to questionnaires in Japanese, and (5) providing informed consent for participation.

In the Japanese healthcare system, ADHD in children is typically diagnosed by physicians, most commonly child psychiatrists and pediatricians with expertise in developmental disorders (8). Current Japanese clinical practice follows nationally developed guidelines for the diagnosis and treatment of ADHD, which adopt DSM-5 criteria (40). Following diagnosis, families may also access developmental disorder support centers (発達障害者支援センター) for ongoing consultation and support services, though these centers serve a supportive rather than diagnostic function. Thus, the term “qualified healthcare professional” in the present study’s inclusion criteria refers to physicians who made the clinical ADHD diagnosis within the Japanese healthcare system.

The sample size of 800 per group was determined through systematic power analysis considerations. Power calculations indicated that a minimum of 176 participants per group would be required to detect small effect sizes (Cohen’s d = 0.3) in mean differences between ADHD and non-ADHD groups with 80% statistical power (α = 0.05, two-tailed). To enable robust subgroup analysis across the three educational stages (elementary, junior high, and senior high school), a minimum of 528 participants per group (176 × 3) would be necessary to maintain adequate power within each stratum. The final sample size of 800 per group was selected to accommodate potential data loss of 10-15% due to incomplete responses or data quality issues while still maintaining sufficient statistical power for both primary comparisons and educational stage-specific analysis.

#### 2.2.2 Sampling strategy

The sampling strategy was based solely on parent-reported ADHD diagnosis status, without intentional demographic balancing within groups. We did not match or stratify the two groups on demographic variables, because methodological evidence—drawn especially from the case-control matching literature—indicates that such matching does not reliably remove confounding and can instead introduce selection and collider bias while requiring that the matching itself be accounted for in the analysis (41, 42). Therefore, participants were enrolled consecutively based on their eligibility criteria and group assignment (ADHD or non-ADHD) without imposed quotas for child grade levels, sex, or geographical distribution. The naturally occurring demographic distribution across Japan’s 47 prefectures and educational stages (elementary: grades 1-6, junior high: grades 7-9, senior high: grades 10-12) was preserved, with demographic variables treated as measured covariates for analytical adjustment rather than design-based constraints.

#### 2.2.3 Data collection

Data collection was conducted entirely online through Rakuten Insight’s secure survey platform. Potential participants received an invitation email with a unique survey link. Upon accessing the survey, participants were presented with detailed information about the study purpose, procedures, data handling, and their rights as research participants. After providing electronic informed consent, participants completed the survey, which took approximately 25–30 minutes.

To ensure data quality, several measures were implemented: response time monitoring prevented progression to subsequent questions if responses were entered too quickly, ensuring adequate time for thoughtful consideration; straight-lining and random responding patterns were identified during post-collection data analysis and removed prior to statistical analysis; and duplicate response prevention through IP address monitoring and panelist ID verification.

### 2.3 Measures

#### 2.3.1 Demographic variables

Comprehensive demographic information was collected from participating parents. Child characteristics included birth-assigned sex (male/female), current age in years, and current grade level (grades 1-12). Parent and family characteristics assessed included the respondent’s birth-assigned sex (male/female) and age in years. Annual household income was measured using an 11-point scale with 1 million yen increments: 1 (less than 1 million yen), 2 (1-1.99 million yen), 3 (2-2.99 million yen), 4 (3-3.99 million yen), 5 (4-4.99 million yen), 6 (5-5.99 million yen), 7 (6-6.99 million yen), 8 (7-7.99 million yen), 9 (8-8.99 million yen), 10 (9-9.99 million yen), and 11 (10 million yen or more). Residential area was categorized as rural, suburban, or urban based on participant self-report.

#### 2.3.2 ADHD symptoms

ADHD Rating Scale-IV (ADHD-RS-IV): The ADHD-RS-IV is an 18-item scale assessing inattention (9 items) and hyperactivity-impulsivity (9 items) symptoms based on DSM-IV criteria (43). Parents rate the frequency of each behavior over the past six months on a 4-point scale (0 = never or rarely, 1 = sometimes, 2 = often, 3 = very often). The Japanese version has been validated in multiple large-scale studies demonstrating excellent psychometric properties and revealing that Japanese children score significantly lower than U.S. children on hyperactive-impulsive symptoms (44–46). In the current sample, the scale demonstrated excellent internal consistency with Cronbach’s α = 0.952 for the inattention subscale, 0.925 for the hyperactivity-impulsivity subscale, and 0.960 for the total score.

#### 2.3.3 Depressive symptoms

Short Mood and Feelings Questionnaire (SMFQ): The SMFQ is a 13-item measure of depressive symptoms in children and adolescents (47). Parents rate how often their child experienced each symptom over the past two weeks on a 3-point scale (0 = not true, 1 = sometimes true, 2 = true). While the Japanese version lacks a standalone validation study, it has demonstrated measurement invariance and a unidimensional factor structure in large Japanese cohorts including the Tokyo Teen Cohort study (48, 49). In the current sample, the scale demonstrated excellent internal consistency (Cronbach’s α = 0.935).

#### 2.3.4 Anxiety symptoms

Spence Children’s Anxiety Scale (SCAS): The SCAS parent-report version consists of 38 items assessing six anxiety domains: separation anxiety (6 items, α = 0.855), social phobia (6 items, α = 0.854), obsessive-compulsive symptoms (6 items, α = 0.906), panic/agoraphobia (9 items, α = 0.950), physical injury fears (5 items, α = 0.761), and generalized anxiety (6 items, α = 0.817) (50, 51). Items are rated on a 4-point scale (0 = never, 1 = sometimes, 2 = often, 3 = always). The Japanese version has been validated with over 2,200 students, confirming the factor structure while revealing lower overall scores and higher obsessive-compulsive symptom endorsement compared to Western samples (52, 53). In the current sample, the total score demonstrated excellent internal consistency (Cronbach’s α = 0.970).

#### 2.3.5 Irritability

Affective Reactivity Index (ARI): The ARI is a 7-item scale measuring irritability in children and adolescents (54). Six items assess the frequency of irritable mood and behaviors (e.g., “Gets angry frequently,” “Often loses temper”) rated on a 3-point scale (0 = not true, 1 = somewhat true, 2 = certainly true), with one item assessing impairment. The Japanese version was recently validated with over 6,400 student-parent pairs, demonstrating comparable internal consistency to the original English version (55). In the current sample, the scale showed excellent internal consistency (Cronbach’s α = 0.904).

#### 2.3.6 Bullying victimization

Parents reported the frequency of their child experiencing bullying over the past two months using a 5-point scale: 1 (never), 2 (once or twice in the past two months), 3 (two or three times a month), 4 (about once a week), and 5 (several times a week). Higher scores indicated greater frequency of bullying victimization.

#### 2.3.7 Academic achievement

Parents rated their child’s overall academic performance using a 5-point scale: 1 (very high, top 1-20%), 2 (somewhat high, top 20-40%), 3 (average, top 40-60%), 4 (somewhat low, top 60-80%), and 5 (very low, top 80-100%). For analyses, scores were reverse-coded so that higher values indicated better academic performance, facilitating interpretation of results.

#### 2.3.8 Social support

Friend Support: Friend support was measured by asking parents “How often do your child’s friends help when your child is in trouble?” with response options ranging from 1 (never help) to 5 (very often help), including 2 (rarely help), 3 (neither often nor rarely), and 4 (often help). Higher scores indicated greater perceived support.

Teacher Support: Teacher support was assessed using an identical question format, substituting “school teachers” for “your child’s friends” in the item stem. Higher scores indicated greater perceived support.

### 2.4 Statistical analysis plan

#### 2.4.1 Data preparation

Prior to analysis, data were screened for careless responding using three detection methods based on recommendations from Ward and Meade (56). Longstring analysis identified straight-lining patterns, defined as 10 or more consecutive identical responses. Mahalanobis distance was calculated to detect multivariate outliers indicating unusual response patterns across multiple variables. Individual Response Variability (IRV) analysis identified responses with zero variability, suggesting insufficient attention to item content. These detection indices were implemented using the careless R package (57).

Critically, the decision to exclude only respondents flagged by all three indices simultaneously—rather than by any single index—was guided by the clinical characteristics of the study population. In research on ADHD and mental health, single-indicator exclusion criteria risk the systematic removal of participants with genuine clinical severity. For example, a parent reporting uniformly high scores on the SMFQ for a child with severe depressive symptoms would produce both a longstring pattern and zero response variability, thereby triggering two of the three detection indices despite providing valid clinical data. The same logic applies to the ADHD-RS-IV, where a parent of a child with severe combined-presentation ADHD may endorse consistently high ratings across inattention and hyperactivity-impulsivity items, and to the ARI, where a child with pronounced irritability may receive uniformly elevated scores. Excluding such respondents on the basis of a single indicator would disproportionately remove the very participants whose clinical profiles are most relevant to the research questions. The conservative intersection criterion—requiring convergence across all three independent detection methods—minimizes the false-positive exclusion of clinically valid responses while retaining sensitivity to genuinely careless responding, consistent with the multi-method triangulation approach recommended by Ward and Meade (56). Responses flagged by all three detection methods were excluded from subsequent analyses to ensure data quality.

#### 2.4.2 Descriptive analyses

Descriptive statistics (means, standard deviations, frequencies, and percentages) were calculated for all demographic variables and mental health measures. Comparisons between the ADHD and non-ADHD groups were conducted for all study variables. For group comparisons, the Wilcoxon rank-sum test was used for continuous variables, while the chi-square test was employed for categorical variables. The first hypothesis, which predicted that children with ADHD would demonstrate significantly poorer outcomes across all measured domains compared to children without ADHD, would be supported if the ADHD group showed higher scores on negative indicators (ADHD symptoms, depressive symptoms, anxiety symptoms, irritability, and bullying victimization) and lower scores on positive indicators (academic achievement, friend support, and teacher support). Correlations among study variables were examined using Pearson’s correlations. To control the false discovery rate (FDR) arising from multiple comparisons, the Benjamini–Hochberg (BH) procedure was applied (58). Study variables were organized into two testing families. Family A comprised seven background demographic comparisons (child age, child sex, school level, respondent age, respondent sex, annual family income, and residence); because these comparisons served a descriptive purpose—characterizing the sample rather than testing substantive hypotheses—no multiplicity adjustment was applied, and this is noted in **Table 1**. Family B comprised the 16 primary outcome comparisons (ADHD symptoms: inattention, hyperactivity-impulsivity, and total score; mental health: depression, separation anxiety, social phobia, obsessive-compulsive, panic/agoraphobia, physical injury fears, generalized anxiety, total anxiety, and irritability; school functioning: bullying victimization, academic achievement, friend support, and teacher support). The BH method was chosen over the Bonferroni correction because the outcome variables exhibited moderate-to-high intercorrelations (r = .48–.72), under which the Bonferroni correction becomes overly conservative (59). Adjusted p-values (q-values) are reported alongside raw p-values in **Table 1** for all Family B comparisons.

**Table 1:** Participant characteristics.

Study variables	Overall N = 1,594¹	ADHD N = 797¹	Non-ADHD N = 797¹	p-value²	q-value³	Effect size^6^
Family A: background variables^4^						
Child Age (years)	13.16 (3.38)	12.93 (3.33)	13.39 (3.42)	0.002	—	d = −0.14 [−0.23, −0.04]
Child Sex				<0.001	—	V = 0.22
Girl	604 (37.9%)	216 (27.1%)	388 (48.7%)			
Boy	990 (62.1%)	581 (72.9%)	409 (51.3%)			
School Level				0.015	—	V = 0.07
Elementary (Grades 1-6)	559 (35.1%)	294 (36.9%)	265 (33.2%)			
Junior high (Grades 7-9)	558 (35.0%)	291 (36.5%)	267 (33.5%)			
Senior high (Grades 10-12)	477 (29.9%)	212 (26.6%)	265 (33.2%)			
Respondent Age (years)	45.58 (6.66)	44.20 (6.71)	46.95 (6.32)	<0.001	—	d = −0.42 [−0.52, −0.32]
Respondent Sex				<0.001	—	V = 0.15
Female	820 (51.4%)	470 (59.0%)	350 (43.9%)			
Male	774 (48.6%)	327 (41.0%)	447 (56.1%)			
Annual Family Income				<0.001	—	d = −0.31 [−0.41, −0.22]
Less than 1M JPY	36 (2.3%)	26 (3.3%)	10 (1.3%)			
1-1.99M JPY	42 (2.6%)	28 (3.5%)	14 (1.8%)			
2-2.99M JPY	79 (5.0%)	50 (6.3%)	29 (3.6%)			
3-3.99M JPY	141 (8.8%)	89 (11.2%)	52 (6.5%)			
4-4.99M JPY	186 (11.7%)	98 (12.3%)	88 (11.0%)			
5-5.99M JPY	213 (13.4%)	114 (14.3%)	99 (12.4%)			
6-6.99M JPY	175 (11.0%)	71 (8.9%)	104 (13.0%)			
7-7.99M JPY	192 (12.0%)	96 (12.0%)	96 (12.0%)			
8-8.99M JPY	148 (9.3%)	76 (9.5%)	72 (9.0%)			
9-9.99M JPY	110 (6.9%)	33 (4.1%)	77 (9.7%)			
10M JPY or more	272 (17.1%)	116 (14.6%)	156 (19.6%)			
Residence				0.002	—	V = 0.09
Rural	283 (17.8%)	164 (20.6%)	119 (14.9%)			
Suburban	833 (52.3%)	418 (52.4%)	415 (52.1%)			
Urban	478 (30.0%)	215 (27.0%)	263 (33.0%)			
Family B: primary outcomes^5^						
ADHD symptoms (ADHD-RS-IV)						
ADHD-RS Total	12.75 (13.19)	21.19 (12.23)	4.30 (7.47)	<0.001	<0.001	d = 1.67 [1.55, 1.78]
ADHD-RS Inattention	8.14 (7.96)	13.35 (7.12)	2.93 (4.66)	<0.001	<0.001	d = 1.73 [1.62, 1.85]
ADHD-RS Hyperactivity	4.61 (6.05)	7.84 (6.48)	1.38 (3.24)	<0.001	<0.001	d = 1.26 [1.15, 1.37]
Depression (SMFQ)	4.21 (5.57)	6.55 (6.16)	1.87 (3.62)	<0.001	<0.001	d = 0.93 [0.82, 1.03]
Anxiety symptoms (SCAS)						
Separation Anxiety	2.45 (3.62)	3.59 (4.20)	1.32 (2.46)	<0.001	<0.001	d = 0.66 [0.56, 0.76]
Social Phobia	2.83 (3.75)	4.12 (4.29)	1.54 (2.53)	<0.001	<0.001	d = 0.73 [0.63, 0.83]
Obsessive-Compulsive	1.96 (3.52)	3.23 (4.19)	0.70 (2.00)	<0.001	<0.001	d = 0.77 [0.67, 0.87]
Panic/Agoraphobia	2.18 (4.79)	3.58 (5.85)	0.77 (2.80)	<0.001	<0.001	d = 0.61 [0.51, 0.71]
Physical Injury Fears	3.02 (3.31)	3.94 (3.70)	2.09 (2.56)	<0.001	<0.001	d = 0.58 [0.48, 0.68]
Generalized Anxiety	3.28 (3.47)	4.73 (3.78)	1.82 (2.37)	<0.001	<0.001	d = 0.92 [0.82, 1.03]
SCAS Total	15.72 (20.16)	23.20 (23.24)	8.25 (12.68)	<0.001	<0.001	d = 0.80 [0.70, 0.90]
Irritability (ARI)	2.32 (3.11)	3.75 (3.48)	0.89 (1.75)	<0.001	<0.001	d = 1.04 [0.93, 1.14]
Bullying Victimization				<0.001	<0.001	V = 0.30
None	1,287 (80.7%)	550 (69.0%)	737 (92.5%)			
Once or twice every 2 months	186 (11.7%)	144 (18.1%)	42 (5.3%)			
2–3 times a month	58 (3.6%)	48 (6.0%)	10 (1.3%)			
About once a week	31 (1.9%)	27 (3.4%)	4 (0.5%)			
Several times a week	32 (2.0%)	28 (3.5%)	4 (0.5%)			
Academic Achievement				<0.001	<0.001	V = 0.37
Low (top 80-100%)	320 (20.1%)	257 (32.2%)	63 (7.9%)			
Below average (top 60-80%)	252 (15.8%)	166 (20.8%)	86 (10.8%)			
Average (top 40-60%)	493 (30.9%)	180 (22.6%)	313 (39.3%)			
High (top 20-40%)	290 (18.2%)	112 (14.1%)	178 (22.3%)			
Very high (top 1-20%)	239 (15.0%)	82 (10.3%)	157 (19.7%)			
Support from Friends				<0.001	<0.001	V = 0.22
Not at all supportive	60 (3.8%)	47 (5.9%)	13 (1.6%)			
Not very supportive	132 (8.3%)	101 (12.7%)	31 (3.9%)			
Neutral	651 (40.8%)	334 (41.9%)	317 (39.8%)			
Supportive	609 (38.2%)	251 (31.5%)	358 (44.9%)			
Very supportive	142 (8.9%)	64 (8.0%)	78 (9.8%)			
Support from Teachers				<0.001	<0.001	V = 0.18
Not at all supportive	51 (3.2%)	33 (4.1%)	18 (2.3%)			
Not very supportive	137 (8.6%)	90 (11.3%)	47 (5.9%)			
Neutral	581 (36.4%)	238 (29.9%)	343 (43.0%)			
Supportive	631 (39.6%)	311 (39.0%)	320 (40.2%)			
Very supportive	194 (12.2%)	125 (15.7%)	69 (8.7%)			

¹ Mean (SD); n (%).

² Wilcoxon rank-sum test; Pearson’s chi-squared test

³ Benjamini–Hochberg adjusted p-value (FDR); applied to Family B comparisons only

^4^ Background comparisons are descriptive; FDR correction not applied

^5^ BH-FDR corrected across 16 primary outcome comparisons

^6^ Effect size for the overall ADHD vs. non-ADHD comparison: Cohen’s d (95% CI) for continuous variables (positive values indicate higher scores in the ADHD group); Cramér’s V for categorical variables.

#### 2.4.3 Inferential analyses

Three-way analysis of variance (ANOVA) were conducted with ADHD status (ADHD vs. non-ADHD), child sex (male vs. female), and school stage (elementary, junior high, senior high) as independent variables for each outcome measure. Main effects, two-way interactions, and three-way interactions were examined. To evaluate the second hypothesis regarding differential effects across educational stages, standardized mean differences (SMD, Cohen’s d) between ADHD and non-ADHD groups were calculated separately for each school stage. The hypothesis would be supported if the group differences were most pronounced during the junior high school period, as evidenced by larger effect sizes for that educational stage. To test the third hypothesis regarding sex-specific patterns, SMDs were calculated separately for males and females. The hypothesis would be supported if effect sizes for the association between ADHD status and internalizing symptoms (depression, anxiety), irritability, and social difficulties were larger for females than males. All analyses were conducted using R version 4.3.0.

To evaluate the robustness of observed group differences after accounting for socioeconomic status, sensitivity analyses were conducted using analysis of covariance (ANCOVA) with annual household income (centered continuous variable) as a covariate. Two-way ANCOVAs examined ADHD status and school stage as fixed factors with household income as a covariate for all 12 outcome variables (six SCAS subscales, SCAS total, SMFQ, academic achievement, bullying victimization, friend support, and teacher support). Three-way ANCOVAs further incorporated child sex as an additional fixed factor for the seven primary outcome composites (depression, total anxiety, irritability, bullying victimization, academic achievement, friend support, and teacher support). The assumption of homogeneity of regression slopes was tested for each model. For two-way ANCOVAs, the income × ADHD status and income × school stage interaction terms were examined. For three-way ANCOVAs, all covariate interaction terms were tested, including income × sex, income × ADHD status, income × school stage, and all higher-order interactions involving the covariate (i.e., income × sex × ADHD, income × sex × school stage, income × ADHD × school stage, and income × sex × ADHD × school stage). All *p*-values from ANCOVA sensitivity analysis were corrected for multiple comparisons using the Benjamini–Hochberg false discovery rate (FDR) procedure.

To examine the differential contributions of ADHD symptom dimensions to functional outcomes, two complementary supplementary analysis were conducted: presentation subtype classification (Grades 1–9 only, n = 585) and symptom-dimension regression analysis (full ADHD sample, N = 797). For subtype classification, participants in Grades 1–9 were classified into ADHD presentation subtypes based on the ADHD Rating Scale-IV Japanese version (43) using 95th percentile cutoffs from Japanese standardization norms (60). Cutoff values were specific to grade, sex, and rater type (parent-report). Participants exceeding the inattention subscale cutoff only were classified as Predominantly Inattentive; those exceeding the hyperactivity/impulsivity cutoff only were classified as Predominantly Hyperactive-Impulsive; and those exceeding both cutoffs were classified as Combined presentation. Participants scoring below both cutoffs were retained in the ADHD group based on their parent-reported diagnosis but were not assigned a presentation subtype. Participants in Grades 10–12 (n = 212) could not be classified because 95th percentile cutoffs for senior high school students have not been established in the Japanese normative data. To further examine the unique contributions of each symptom dimension, multiple regression analyses were conducted within the entire ADHD group (N = 797), with inattention and hyperactivity/impulsivity subscale scores entered as simultaneous predictors for each outcome variable. False discovery rate (FDR) correction (58) was applied across all 28 regression coefficients (14 outcome models × 2 predictors) to control for multiple comparisons; FDR-adjusted q values are reported alongside p values.

#### 2.4.4 Ethical considerations

This study received approval from the Shinshu University Ethics Committee on Educational Research (Approval number: 20-25). All participants provided electronic informed consent before participating. The consent form explicitly stated that participation was voluntary, that participants could withdraw at any time without penalty, and that all data would be anonymized and used only for research purposes. Participants received compensation in the form of points through the Rakuten Insight platform (equivalent to approximately 300 JPY) upon survey completion, consistent with standard panel participation rates. This modest compensation was designed to acknowledge participants’ time contribution while maintaining the voluntary nature of participation.

## 3 Results

### 3.1 Data quality and sample characteristics

#### 3.1.1 Careless response detection

Prior to analysis, data quality screening identified potential careless responding using three detection indices. Of the initial 1,600 participants, longstring analysis detected 1,217 cases (76.1%) with 10 or more consecutive identical responses, Mahalanobis distance identified 373 cases (23.3%) with p <.001 indicating multivariate outliers, and individual response variability analysis detected 438 cases (27.4%) with zero variability. Six participants (0.38%) were flagged by all three indices and excluded from subsequent analysis, resulting in a final analytical sample of 1,594 participants (797 in the ADHD group, 797 in the non-ADHD group).

#### 3.1.2 Participant characteristics

**Table 1** presents the demographic and clinical characteristics of the sample. The ADHD and non-ADHD groups differed significantly on several demographic variables. The ADHD group had a markedly higher proportion of boys (73% vs. 51%, χ² = 77.95, *p* <.001), reflecting the well-documented male predominance in ADHD diagnoses. Mothers constituted a larger proportion of respondents in the ADHD group than in the non-ADHD group (59% vs. 44%, χ² = 35.57, *p* <.001). Parents of children with ADHD were younger (*M* = 44.20, *SD* = 6.71 vs. *M* = 46.95, *SD* = 6.32 years, *p* <.001) and reported lower annual household income (*M* = 6.67 vs. *M* = 7.52 on the 11-point scale, *p* <.001) compared to non-ADHD parents; the income difference was subsequently addressed through ANCOVA sensitivity analysis (see Sensitivity Analysis section below). The distribution across educational stages also differed between groups (χ² = 8.43, *df* = 2, *p* = .015): the ADHD group had a somewhat higher proportion of elementary (37% vs. 33%) and junior high school students (37% vs. 34%) but a lower proportion of senior high school students (27% vs. 33%) compared to the non-ADHD group.

### 3.2 Hypothesis 1: group differences across all domains

As predicted, children with ADHD demonstrated significantly poorer outcomes across all measured domains compared to children without ADHD (**Table 1**). The ADHD group showed markedly elevated ADHD symptoms on the ADHD-RS-IV, with mean scores for inattention (13.35 vs. 2.93), hyperactivity-impulsivity (7.84 vs. 1.38), and total symptoms (21.19 vs. 4.30), all significantly higher than non-ADHD children.

Mental health symptoms were substantially elevated in the ADHD group. Depression scores on the SMFQ were 3.5 times higher (6.55 vs. 1.87), total anxiety scores on the SCAS were nearly three times higher (23.20 vs. 8.25), and irritability scores on the ARI were over four times higher (3.75 vs. 0.89) compared to non-ADHD children. All anxiety subscales showed similar patterns, with separation anxiety (3.59 vs. 1.32), social phobia (4.12 vs. 1.54), obsessive-compulsive symptoms (3.23 vs. 0.70), panic/agoraphobia (3.58 vs. 0.77), physical injury fears (3.94 vs. 2.09), and generalized anxiety (4.73 vs. 1.82) all significantly elevated in the ADHD group.

School functioning showed consistent impairment in the ADHD group. Bullying victimization was substantially higher, with 31.0% of children with ADHD experiencing some form of bullying compared to only 7.5% of non-ADHD children. Academic achievement was significantly lower, with 53.0% of the ADHD group performing below average (bottom 60%) compared to only 18.7% of non-ADHD children. Social support was also compromised, with children with ADHD receiving less support from both friends and teachers, though the teacher support difference was less pronounced.

### 3.3 Correlations among study variables

**Figure 1** displays the correlation matrix among all study variables. Strong positive correlations emerged among symptom measures, with the highest correlations observed between inattention and hyperactivity (r = .77, p <.001), depression and anxiety (r = .72, p <.001), and hyperactivity and irritability (r = .66, p <.001). ADHD symptoms showed moderate to strong positive correlations with all mental health indicators (rs = .48–.66, all ps <.001, two-tailed) and bullying victimization (rs = .34–.40, all ps <.001, two-tailed), while showing negative correlations with academic achievement (rs = −.25 to −.46, all ps <.001, two-tailed).

**Figure 1:**
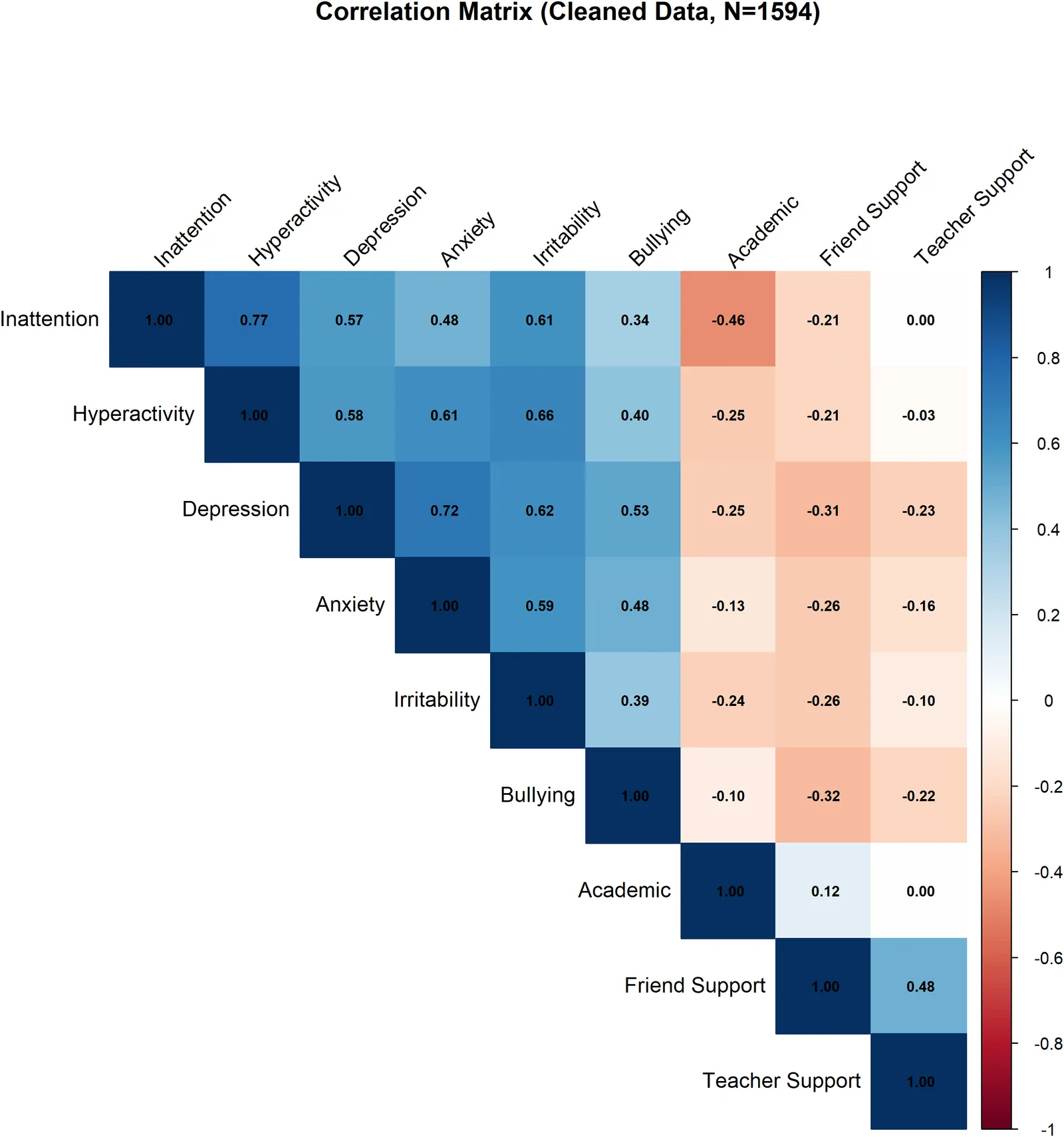
Correlation matrix of study variables. Pearson correlations among ADHD symptoms (inattention, hyperactivity-impulsivity), mental health indicators (depression, anxiety, irritability), school functioning measures (bullying victimization, academic achievement), and social support variables (friend support, teacher support) for the total sample (N = 1,594). Color intensity indicates correlation strength, with blue indicating positive correlations and red indicating negative correlations.

Mental health symptoms were strongly intercorrelated, with depression, anxiety, and irritability showing correlations ranging from .59 to .72 (all ps <.001, two-tailed). All mental health symptoms were positively associated with bullying victimization (rs = .39–.53, all ps <.001, two-tailed) and negatively associated with friend support (rs = −.26 to −.31, all ps <.001, two-tailed) and teacher support (rs = −.10 to −.23, all ps <.001, two-tailed). Academic achievement showed weak to moderate negative correlations with all symptom measures (rs = −.10 to −.46, all ps <.001, two-tailed).

### 3.4 Hypotheses 2 and 3: educational stage and sex effects

#### 3.4.1 Main effects and interactions

Three-way ANOVAs examining ADHD status, child sex, and school stage revealed complex patterns of main effects and interactions (**Supplementary Tables 1**, **2**). Significant main effects of ADHD status emerged for all outcomes except teacher support (F = 2.38, p = .123). Sex main effects were significant for depression (F = 17.32, p <.001), anxiety (F = 47.36, p <.001), irritability (F = 7.23, p = .007), bullying victimization (F = 9.84, p = .002), academic achievement (F = 5.67, p = .017), and teacher support (F = 10.57, p = .001). School stage main effects were significant for depression (F = 5.56, p = .004), anxiety (F = 10.16, p <.001), irritability (F = 5.03, p = .007), bullying victimization (F = 8.85, p <.001), and academic achievement (F = 7.96, p <.001).

Critically, significant ADHD × sex interactions emerged for depression (F = 8.53, p = .004), anxiety (F = 18.19, p <.001), irritability (F = 7.06, p = .008), and bullying victimization (F = 8.79, p = .003), supporting the hypothesis that ADHD-related difficulties manifest differently by sex. ADHD × school stage interactions were significant for depression (F = 4.75, p = .009), anxiety (F = 5.02, p = .007), bullying victimization (F = 4.26, p = .014), and academic achievement (F = 3.25, p = .039), indicating that the impact of ADHD varies across educational stages. No significant three-way interactions were observed.

#### 3.4.2 Sensitivity analysis

To assess whether household income confounded the observed ADHD-related group differences, two-way ANCOVAs were conducted with annual household income (centered) as a covariate. The assumption of homogeneity of regression slopes was satisfied for all outcomes, as neither the income × ADHD status interactions (all *q*s >.730) nor the income × school stage interactions (all *q*s >.117) reached significance after FDR correction. After controlling for household income, the main effect of ADHD status remained significant for all outcomes except teacher support, *F*(1, 1587) = 0.60, *p* = .439, which was also nonsignificant in the primary ANOVA. Notably, the ADHD main effects retained large *F*-values: depression, *F*(1, 1587) = 307.91, *p* <.001; total anxiety, *F*(1, 1587) = 227.72, *p* <.001; academic achievement, *F*(1, 1587) = 158.46, *p* <.001; bullying victimization, *F*(1, 1587) = 111.89, *p* <.001; and friend support, *F*(1, 1587) = 52.40, *p* <.001 (all *q*s <.001). Household income emerged as a significant covariate for 4 of 12 outcomes after FDR correction: academic achievement, *F*(1, 1587) = 34.93, *q* <.001; physical injury fears, *F*(1, 1587) = 9.55, *q* = .012; separation anxiety, *F*(1, 1587) = 5.82, *q* = .048; and social phobia, *F*(1, 1587) = 6.57, *q* = .042. Income was not a significant covariate for depression (*q* = .140), irritability, bullying victimization, obsessive-compulsive symptoms, panic/agoraphobia, generalized anxiety, total anxiety, friend support, or teacher support (all *q*s >.053). The ADHD status × school stage interactions also remained significant after income adjustment for depression, *F*(2, 1587) = 3.79, *q* = .039; total anxiety, *F*(2, 1587) = 5.19, *q* = .011; bullying victimization, *F*(2, 1587) = 8.80, *q* <.001; and academic achievement, *F*(2, 1587) = 12.32, *q* <.001.

Three-way ANCOVAs incorporating child sex, ADHD status, school stage, and household income were similarly examined for homogeneity of regression slopes. None of the seven covariate interaction terms (income × sex, income × ADHD, income × school stage, income × sex × ADHD, income × sex × school stage, income × ADHD × school stage, and income × sex × ADHD × school stage) reached significance for any outcome after FDR correction (all *q*s >.107), confirming that the assumption was satisfied across all models. These three-way ANCOVAs confirmed that the critical ADHD × sex interaction patterns were preserved after income adjustment: depression, *F*(1, 1581) = 8.22, *q* = .010; total anxiety, *F*(1, 1581) = 17.61, *q* <.001; irritability, *F*(1, 1581) = 7.04, *q* = .014; and bullying victimization, *F*(1, 1581) = 8.85, *q* = .010. The ADHD × school stage interactions also remained significant for depression, *F*(2, 1581) = 4.76, *q* = .030; total anxiety, *F*(2, 1581) = 4.93, *q* = .030; and bullying victimization, *F*(2, 1581) = 4.24, *q* = .034. No significant three-way interactions emerged. In the three-way ANCOVAs, household income was a significant covariate only for academic achievement, *F*(1, 1581) = 35.47, *q* <.001, and did not reach significance for any other outcome after FDR correction (all *q*s >.086).

#### 3.4.3 ADHD symptom dimension analysis

Among the 585 ADHD group participants in Grades 1–9 eligible for subtype classification, 238 (40.7%) scored below both subscale cutoffs, 184 (31.5%) met criteria for Combined presentation, 90 (15.4%) for Predominantly Inattentive presentation, and 73 (12.5%) for Predominantly Hyperactive-Impulsive presentation. The remaining 212 participants in Grades 10–12 were designated as cutoff unavailable due to the absence of Japanese normative data for senior high school students.

Turning from subtype classification (Grades 1–9) to symptom-dimension analysis across the full ADHD sample, multiple regression analysis (N = 797, including Grades 10–12) revealed distinct functional profiles for the two symptom dimensions. All models had acceptable collinearity (VIF = 1.62 for all predictors). Hyperactivity/impulsivity emerged as the dominant predictor across mental health and social outcomes, with the largest standardized coefficients observed for separation anxiety (β = .60, q <.001), panic/agoraphobia (β = .58, q <.001), total anxiety (β = .58, q <.001), obsessive-compulsive symptoms (β = .57, q <.001), generalized anxiety (β = .50, q <.001), irritability (β = .46, q <.001), physical injury fears (β = .44, q <.001), and social phobia (β = .37, q <.001). Hyperactivity/impulsivity also significantly predicted depression (β = .34, q <.001), bullying victimization (β = .32, q <.001), and reduced friend support (β = −.13, q = .006). Models for mental health outcomes explained 10–31% of variance (R² = .10–.31).

In contrast, inattention was the strongest predictor of academic impairment (β = −.51, q <.001), representing the largest single standardized coefficient across all models. After controlling for inattention, hyperactivity/impulsivity showed a positive association with academic achievement (β = .25, q <.001) despite a near-zero bivariate correlation (r = −.07), a pattern consistent with a statistical suppression effect. Inattention also independently predicted depression (β = .20, q <.001) and irritability (β = .14, q <.001). Notably, after controlling for hyperactivity/impulsivity, inattention showed small negative associations with several anxiety subscales—separation anxiety (β = −.13, q = .001), obsessive-compulsive symptoms (β = −.15, q <.001), panic/agoraphobia (β = −.19, q <.001), and total anxiety (β = −.08, q = .049)—despite positive zero-order correlations, a pattern that may reflect suppression effects arising from shared variance with hyperactivity/impulsivity. Neither symptom dimension significantly predicted teacher support (model p = .24).

#### 3.4.4 Effect sizes across educational stages

**Figure 2** and **Supplementary Table 3** present standardized mean differences (Cohen’s d) between ADHD and non-ADHD groups across educational stages and sex. Contrary to our hypothesis that differences would be most pronounced in junior high school, effect sizes showed variable patterns across outcomes and stages.

**Figure 2:**
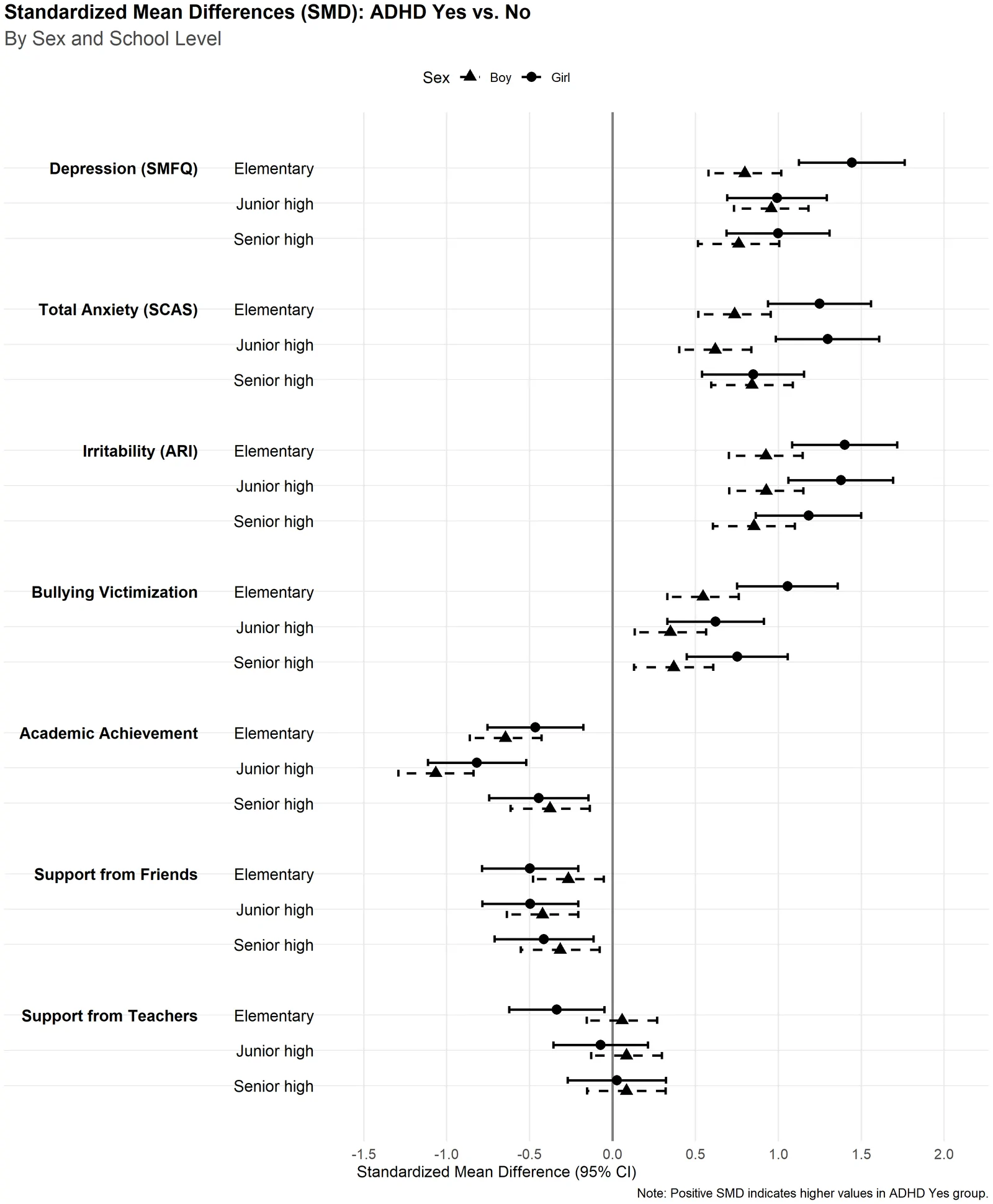
Standardized mean differences between ADHD and non-ADHD groups across educational stages and child sex. Cohen’s d effect sizes comparing children with ADHD to non-ADHD children on seven outcome measures, stratified by educational stage (elementary, junior high, senior high school) and child sex. Positive values indicate higher scores in the ADHD group, while negative values indicate lower scores in the ADHD group. Error bars represent 95% confidence intervals.

For mental health outcomes, depression effect sizes for girls were largest in elementary school (d = 1.41) and somewhat smaller in junior high (d = 0.99) and senior high school (d = 1.00), whereas for boys they were relatively stable across stages, with the largest effect in junior high school (elementary: d = 0.80; junior high: d = 0.96; senior high: d = 0.76). For girls, anxiety effect sizes were comparably large in elementary (d = 1.25) and junior high school (d = 1.30) and smaller in senior high school (d = 0.82), whereas for boys, the smallest effect was observed in junior high school (d = 0.63) and the largest in senior high school (d = 0.84; elementary: d = 0.74). Irritability effects were consistently large across all stages, with particularly strong effects for girls (ds = 1.18-1.40) compared to boys (ds = 0.85-0.93).

Bullying victimization effects were most pronounced in elementary school for both sexes (girls: d = 1.06, boys: d = 0.55) and decreased substantially by junior high school (girls: d = 0.62, boys: d = 0.35), with slight increases in senior high school. Academic achievement showed the opposite pattern, with effect sizes increasing from elementary to junior high school, particularly for boys (elementary: d = 0.65, junior high: d = 1.07, senior high: d = 0.38).

#### 3.4.5 Sex-specific patterns

Supporting our third hypothesis, girls with ADHD showed consistently larger effect sizes than boys across most mental health domains. The sex difference was particularly pronounced for depression (mean d for girls = 1.14 vs. boys = 0.84), anxiety (girls = 1.13 vs. boys = 0.73), and irritability (girls = 1.32 vs. boys = 0.90). Girls also showed larger effects than boys for bullying victimization at all three stages (elementary: d = 1.06 vs. 0.55; junior high: d = 0.62 vs. 0.35; senior high: d = 0.75 vs. 0.37)

For academic achievement and social support, sex differences were less consistent. Boys showed larger effect sizes for academic impairment in junior high school (d = 1.07 vs. 0.82 for girls), while girls showed larger effects for friend support deficits across all stages. Teacher support showed minimal group differences regardless of sex or educational stage, with most effect sizes below 0.4.

## 4 Discussion

This study aimed to examine mental health symptoms and school functioning in children with and without ADHD across three educational stages—elementary, junior high, and senior high school—within a large Japanese sample. Our first hypothesis was strongly supported: children with ADHD demonstrated significantly elevated depression, anxiety, and irritability symptoms, higher bullying victimization, poorer academic performance, and reduced social support compared to non-ADHD peers, with effect sizes ranging from moderate to very large across domains. Our second hypothesis regarding stage-specific patterns received partial support, though contrary to expectations, the largest group differences for girls' depression emerged in elementary rather than junior high school, and girls' anxiety differences were comparably large in elementary and junior high school while academic difficulties peaked in junior high as anticipated. Our third hypothesis was confirmed, with girls showing consistently larger effect sizes than boys for internalizing symptoms and irritability across all educational stages. These findings contribute to understanding of developmental and sex-specific patterns in the relationship between ADHD and functional outcomes across educational contexts. Importantly, beyond replicating established associations in a non-Western sample, the present study extends prior research in three specific ways. First, whereas previous Japanese ADHD research has focused primarily on adult comorbidity rates (12) or population-level medication prevalence (38), this study provides a multi-domain characterization of ADHD-related impairments spanning three educational stages in a cultural context where medication rates remain among the lowest internationally (0.4% vs. 5.3% in the United States; 38), ranking among the lowest across 13 countries surveyed (61), and where the high rate of comorbid ASD in diagnosed children (54.4%; 39) may reflect diagnostic practices that prioritize autism spectrum features. Second, by simultaneously examining sex and educational stage, the present data reveal that girls with ADHD bear a a disproportionate internalizing burden that is most pronounced before senior high school (depression: d = 1.41 in elementary school; anxiety: d = 1.25 and 1.30 in elementary and junior high school, respectively) — a sex × stage interaction pattern that, to our knowledge, has not been jointly quantified across the elementary-to-senior-high trajectory. Third, the symptom dimension analysis demonstrate a functional dissociation within the ADHD group whereby hyperactivity/impulsivity primarily predicts emotional and social difficulties (β = .32–.60) while inattention selectively drives academic impairment (β = −.51), extending meta-analytic findings that have examined these associations in separate samples (37, 62).

### 4.1 ADHD-related impairments across all domains

Consistent with our first hypothesis, children with ADHD demonstrated significantly poorer outcomes across nearly all measured domains compared to their non-ADHD peers, with teacher support the sole exception. Although the teacher support difference was significant in the unadjusted comparison (**Table 1**), the ADHD main effect on teacher support was not statistically significant in the three-way analysis (F = 2.38, p = .123) and was the smallest in magnitude, so this domain warrants cautious interpretation. The magnitude of these differences was substantial, with effect sizes ranging from moderate to very large across mental health, social, and academic domains. The elevation in ADHD symptoms was expected by definition, but the striking 3- to 4-fold increases in depression, anxiety, and irritability scores align with international meta-analysis documenting high rates of emotional comorbidities in ADHD (10, 17). Meta-analytic evidence from Tung et al. (31) found that girls with ADHD were 3.2 times more likely to have anxiety disorders and 4.2 times more likely to have depression compared to non-ADHD girls. These findings extend previous Japanese research that found dramatic elevations in adult ADHD comorbidity rates—60-fold higher depression and 40-fold higher anxiety compared to controls (12)—by demonstrating that such patterns emerge early in childhood and persist across educational stages.

Importantly, Hypothesis 1 was formulated as an *a priori* confirmatory hypothesis grounded in extensive prior evidence rather than as an exploratory proposition. The World Federation of ADHD International Consensus Statement, synthesizing findings across 208 evidence-based conclusions, established that ADHD is associated with impairment across academic, social, and emotional domains simultaneously (63). Our broad hypothesis was thus theoretically motivated, and its comprehensive scope was deliberately designed to test whether these well-documented multi-domain impairments replicate in a Japanese population—a cultural context where ADHD recognition and management differ substantially from Western settings (38). The breadth of Hypothesis 1 therefore represents a methodological strength for cross-cultural validation, enabling systematic comparison of domain-specific effect sizes with international findings rather than selectively testing a subset of outcomes.

The finding that 31% of children with ADHD experienced bullying victimization compared to only 7.5% of non-ADHD children represents a critical concern for educational and clinical practice. This four-fold increase aligns with previous research documenting that elevated rates (57%) of children with ADHD experiences weekly peer victimization in middle school (64). The strong correlations between ADHD symptoms and bullying victimization (rs = .34-.40) indicate that both core symptoms contribute to peer difficulties, with hyperactivity-impulsivity identified as the primary risk factor for victimization. These findings align with transactional developmental models proposing bidirectional relationships between ADHD symptoms and peer difficulties. Tseng et al. (65) demonstrated that inattention specifically predicted subsequent peer impairment through impaired ability to learn social cues and respond appropriately in social situations. This mechanism may be particularly problematic in Japanese educational contexts, where collectivist emphasis on group harmony requires subtle social navigation skills that ADHD-related inattention directly compromises, creating cascading social failures for affected children.

Academic achievement showed expected impairments, with 53% of the ADHD group performing below average compared to 18.7% of controls. This finding corroborates cross-cultural evidence that ADHD-related academic difficulties transcend educational systems (22), with longitudinal research demonstrating that high inattention levels in elementary school are associated with significantly lower educational attainment (37). Despite the structured, supportive nature of Japanese elementary education, children with ADHD face substantial academic challenges that persist across developmental stages.

Sensitivity analyses using ANCOVA with household income as a covariate demonstrated that the observed ADHD-related impairments were robust after accounting for socioeconomic differences between groups. Despite significantly lower household income in the ADHD group (*M* = 6.67 vs. 7.52 on the 11-point scale, *p* <.001), ADHD main effects remained significant for all outcomes except teacher support, and the critical ADHD × sex interactions for depression, anxiety, irritability, and bullying victimization were preserved. Household income emerged as a significant covariate for only 4 of 12 outcomes—academic achievement, physical injury fears, separation anxiety, and social phobia—indicating that socioeconomic confounding was limited to specific domains rather than pervasive across all measured outcomes. The finding that income was most strongly associated with academic achievement is consistent with meta-analytic evidence linking socioeconomic status to academic achievement (66).

The symptom dimension analysis revealed a clear dissociation between the functional correlates of hyperactivity/impulsivity and inattention within the ADHD group. Hyperactivity/impulsivity was the dominant predictor of emotional and social difficulties, with standardized coefficients ranging from .32 to .60 across anxiety subscales, depression, irritability, and bullying victimization, and a smaller but significant negative association with friend support (β = −.13). This pattern is consistent with meta-analytic evidence linking hyperactivity/impulsivity to emotional dysregulation (17, 62) and supports theoretical models positioning this symptom dimension as a broad vulnerability factor for psychosocial impairment. The possible suppression effects observed for inattention on anxiety subscales suggest that the apparent inattention–anxiety association in bivariate analyses may be largely attributable to shared variance with hyperactivity/impulsivity rather than a direct relationship. In contrast, inattention emerged as the primary driver of academic impairment (β = −.51), consistent with longitudinal evidence that high inattention levels in elementary school predict significantly lower educational attainment (37) and cross-cultural findings linking ADHD-related academic difficulties to attention deficits across educational systems (22). These differential profiles carry direct implications for intervention: hyperactivity/impulsivity-dominant presentations may benefit most from emotion regulation and social skills training, whereas inattention-dominant presentations require targeted academic support strategies including organizational skills training and classroom accommodations.

### 4.2 Developmental patterns across educational stages

Our second hypothesis regarding stage-specific patterns received partial support, with distinct developmental trajectories emerging across outcome domains. Contrary to our expectation that junior high school would show the most pronounced group differences, patterns varied considerably by outcome type. For mental health symptoms, the largest ADHD-related differences among girls appeared in elementary school for depression (d = 1.41) and in elementary (d = 1.25) and junior high school (d = 1.30) for anxiety. This unexpected finding may reflect the nature of parent-reported assessments rather than true developmental patterns. The apparent decrease in effect sizes during adolescence may represent what has been described as a “hidden burden” phenomenon (67), where adolescent internalizing symptoms become less visible to parents as children spend less time at home and develop greater emotional autonomy. Critically, the reduction in effect sizes during senior high school admits at least three non-mutually-exclusive interpretations: (a) genuine developmental improvement in ADHD-related difficulties as adolescents acquire compensatory strategies, (b) dilution of the ADHD group by individuals whose symptoms have remitted since the original diagnosis, given that our cross-sectional design cannot distinguish currently symptomatic from remitted cases, and (c) increasing underestimation of difficulties through parent reports as adolescents conceal internalizing symptoms from caregivers (68). The relative contribution of each mechanism cannot be disentangled with the present cross-sectional data, and future longitudinal research with multi-informant assessment is needed to clarify the pattern.

Empirical support for this interpretation comes from studies documenting substantial parent-adolescent discrepancies in ADHD assessment. Sibley et al. (68) found that 70% of adolescents met ADHD diagnostic criteria through combined informant reports, but an additional 17% had clinically significant impairment not captured by standard criteria, demonstrating that symptoms become less observable to parents during adolescence. Parents may be more attuned to their elementary-aged children’s emotional states due to greater daily interaction and children’s less developed emotion regulation strategies that make distress more observable. In contrast, adolescents with ADHD may internalize their struggles, masking depression and anxiety from parental observation while experiencing significant subjective distress. This interpretation aligns with research documenting discrepancies between parent and self-reports of internalizing symptoms that widen during adolescence (69), suggesting that the true burden of mental health problems in adolescents with ADHD may be underestimated in parent-report studies.

The pattern for bullying victimization, with peak effect sizes in elementary school (girls: d = 1.06, boys: d = 0.55) that diminished in junior high school, suggests developmental changes in peer dynamics. This may reflect the development of more sophisticated social skills in adolescence, increased tolerance for individual differences as peers mature, or survivor effects whereby the most severely affected children with ADHD have already exited mainstream educational settings. The slight increase in senior high school warrants attention, potentially reflecting renewed social challenges as academic competition intensifies in preparation for university entrance examinations.

Academic achievement showed the anticipated pattern of increasing difficulties from elementary to junior high school, particularly for boys (d = 0.65 to 1.07). This aligns with research documenting that the transition to multiple teachers and increased organizational demands in secondary school creates particular challenges for students with ADHD (4). The subsequent decrease in effect sizes during senior high school (d = 0.38 for boys) likely reflects selection effects, as Japanese students with severe ADHD-related academic impairments may not progress to academically-oriented senior high schools, instead entering vocational tracks or leaving formal education. Indeed, international research shows that adolescents with ADHD have dropout rates over eight times higher than their peers (24).

### 4.3 Sex differences in ADHD expression and impact

Our third hypothesis regarding sex-specific patterns received strong support, with girls showing consistently larger effect sizes for mental health outcomes across all educational stages. The sex disparities were particularly pronounced for depression (mean d: girls = 1.14 vs. boys = 0.84), anxiety (girls = 1.13 vs. boys = 0.73), and irritability (girls = 1.32 vs. boys = 0.90). These findings are consistent with population-based evidence documenting higher rates of internalizing comorbidities in females with ADHD, with a Danish national cohort study revealing that 11.43% of females with ADHD experienced comorbid depression compared to only 3.25% of males (70).

The larger effect sizes for girls across mental health domains may reflect multiple intersecting factors. The substantial underdiagnosis of ADHD in Japanese females means that girls who receive ADHD diagnoses likely represent more severe cases with greater comorbidity. The female ADHD phenotype, characterized by predominant inattentive symptoms rather than hyperactivity-impulsivity (71), may be particularly impairing in educational contexts that emphasize quiet concentration and detail-oriented work. Another possibility is that the gendered expectations in Japanese society, where girls face stronger pressures for social conformity and academic diligence, may create additional stress when ADHD symptoms interfere with meeting these expectations.

The finding that girls with ADHD showed larger effects for bullying victimization at all three educational stages aligns with research on relational aggression patterns. Studies demonstrate that pathways from ADHD symptoms through peer dislike and victimization to depression are present in girls but not boys (36). Girls with ADHD may be particularly vulnerable to subtle forms of social exclusion that characterize female peer victimization, and their executive function deficits may impair navigation of the complex social hierarchies in Japanese schools.

### 4.4 Theoretical and clinical implications

These findings advance theoretical understanding of ADHD as a developmental disorder with variable expression across contexts and sexes. The strong intercorrelations among symptom measures (rs = .48-.77) support dimensional models of psychopathology suggesting that ADHD represents a vulnerability factor for broader emotional and behavioral dysregulation rather than a discrete categorical condition. Multiple taxometric analyses have supported ADHD’s dimensional structure, with dimensional models demonstrated stronger validity coefficients than categorical models (72). The particularly high correlation between hyperactivity and irritability (r = .66) aligns with emerging research on emotional dysregulation as a core feature of ADHD, with meta-analysis documenting emotion dysregulation in 25-50% of children and 30-70% of adults with ADHD (17). Recent meta-analytic evidence found large effect sizes for emotion dysregulation in ADHD (Hedges’ g = 1.17), particularly for emotional lability (g = 1.20), supporting these features as definitive aspects of ADHD psychopathology (62).

Beyond these theoretical considerations, the present findings offer three contributions that, to our knowledge, have not been jointly addressed in comparable prior studies. The first concerns cultural and systemic context. Japan’s ADHD medication rate of 0.4% is among the lowest across 13 countries surveyed (61), and the high ASD comorbidity rate of 54.4% in diagnosed children (39) may indicate that clinical recognition of ADHD-specific impairments is complicated by co-occurring autism spectrum features. Against this backdrop, our data demonstrate that ADHD-related difficulties in mental health, peer relationships, and academic functioning are pervasive and substantial (effect sizes ranging from moderate to very large) across all three educational stages. Previous Japanese research documented dramatically elevated comorbidity rates in adults with ADHD (12), but the present study extends this evidence developmentally by showing that multi-domain impairments are already evident in elementary school and persist — with domain-specific variation — through senior high school. The second contribution is the joint examination of sex and educational stage. Girls with ADHD exhibited consistently larger effect sizes for depression (mean *d* = 1.14 vs. 0.84 for boys), anxiety (1.13 vs. 0.73), and irritability (1.32 vs. 0.90), with the largest disparities concentrated in elementary school. Although sex differences in ADHD comorbidity have been reported in Nordic registry studies (70) and the female ADHD phenotype has been characterized in clinical samples (71), these lines of evidence appear not to have been integrated with stage-specific effect size comparisons spanning the full elementary-to-senior-high range. The resulting pattern — a disproportionate burden that is most visible in younger girls and diminishes in parent reports during adolescence — reinforces the need for early, sex-specific screening. The third contribution is the within-sample demonstration of a functional dissociation between ADHD symptom dimensions. Hyperactivity/impulsivity emerged as the dominant predictor of emotional and social outcomes (β = .32–.60), whereas inattention was the primary driver of academic impairment (β = −.51), with possible suppression effects suggesting that bivariate inattention–anxiety associations may be largely attributable to shared variance with hyperactivity/impulsivity. Meta-analyses have linked these symptom dimensions to distinct outcome domains individually (17, 37, 62), but their simultaneous estimation within a single educationally diverse sample clarifies their relative magnitudes and offers directly actionable guidance for differentiated intervention planning.

From a clinical perspective, our results highlight the critical need for comprehensive assessment and intervention approaches that address the full spectrum of ADHD-related impairments. The high rates of depression, anxiety, and irritability in children with ADHD indicate that mental health screening should be routine in ADHD assessment, particularly for girls who showed the most pronounced emotional difficulties. The stage-specific patterns suggest that intervention timing and targets should be developmentally informed, with elementary school interventions focusing on emotional support and bullying prevention, while junior high school interventions emphasize organizational skills and academic support.

The minimal group differences in teacher support across all stages raise concerns about educator awareness of ADHD-related challenges in Japanese schools. Unlike friend support, which showed moderate effect sizes, teacher support showed negligible differences between groups, suggesting that teachers may not be providing additional support to students with ADHD despite their greater needs. Research demonstrates that while teacher training programs produce large initial effects for ADHD knowledge (SMD = 1.96), these gains are not maintained at follow-up (73). This finding underscores the importance of ongoing teacher education about ADHD and training in evidence-based classroom accommodations, particularly given the low rates of medication treatment in Japan that place greater emphasis on environmental supports.

### 4.5 Limitations and future directions

Several limitations should be considered when interpreting these findings. First, the cross-sectional design precludes causal inferences about developmental trajectories, and the observed stage differences may reflect cohort effects or selection biases rather than true developmental changes. Longitudinal research following the same children across educational transitions is essential to clarify developmental patterns. Second, reliance on parent-reported ADHD diagnosis without independent verification may introduce classification errors, though the clear group differences across multiple validated measures suggest meaningful diagnostic distinctions. Importantly, because our survey assessed lifetime ADHD diagnosis without recording the timing of diagnosis or current symptom status, the ADHD group may include individuals whose symptoms have partially or fully remitted since the original diagnosis, potentially attenuating group differences—particularly in the senior high school subgroup where effect sizes were smallest. Third, the online survey methodology, while enabling large-scale data collection, may underrepresent families without internet access or technological literacy, potentially limiting generalizability.

Although ANCOVA sensitivity analysis demonstrated that ADHD-related group differences were robust after controlling for household income, mediation analyses examining potential causal pathways were not conducted. The cross-sectional design of this study precludes establishing temporal precedence among variables, rendering causal mediation models inappropriate. Simulation studies have demonstrated that cross-sectional mediation analyses produce substantially biased estimates of indirect effects, frequently yielding misleading conclusions about causal processes even when assumptions appear to be met (74, 75). Future longitudinal research following children across educational transitions is needed to disentangle the temporal ordering of ADHD symptoms, socioeconomic factors, and functional outcomes, and to evaluate potential mediating mechanisms through which ADHD confers risk for emotional and social difficulties across development.

Regarding data quality, individual careless response detection indices flagged substantial proportions of respondents when applied independently: 76.1% for longstring analysis, 27.4% for zero individual response variability, and 23.3% for multivariate outliers. However, these high individual flagging rates may largely reflect the clinical nature of the sample rather than pervasive data unreliability. Parents reporting on children with severe psychopathology would be expected to produce response patterns—such as consistently elevated scores across symptom items—that mimic careless responding on any single detection index, and single-indicator flags may therefore have captured clinically severe but valid responding rather than inattentive participation. Only six participants (0.38%) were flagged by the intersection of all three independent indices, suggesting that a substantial proportion of individually flagged responses may represent valid clinical data rather than careless responding. This conservative exclusion threshold was adopted to preserve the clinical representativeness of the sample, which is essential in research examining the full severity spectrum of ADHD-related difficulties. Importantly, the robustness of our findings is supported by the consistent pattern of large, statistically significant group differences across multiple independently validated measures (ADHD-RS-IV, SMFQ, SCAS, ARI, and school functioning scales), all of which showed coherent effect sizes in the expected directions—a pattern that would be unlikely to emerge from systematically unreliable data.

A critical limitation is the absence of individual-level data on comorbid conditions. Given that ASD is the most common comorbidity in Japanese children with ADHD, affecting 54.4% of diagnosed cases (39), and that neurodevelopmental conditions independently elevate bullying victimization risk—with meta-analytic evidence documenting elevated prevalence in youth with ASD (76) and across neurodevelopmental and psychiatric conditions more broadly (77)—the observed associations between ADHD status and bullying victimization or academic difficulties may partly reflect unmeasured confounding by comorbid ASD or learning disabilities rather than ADHD-specific effects. The population-based recruitment through Rakuten Insight enhances external validity relative to clinic-referred samples; however, without structured diagnostic interviews or systematic assessment of comorbid conditions, the extent to which our findings are attributable to ADHD per se versus co-occurring neurodevelopmental conditions cannot be determined. Future research should employ structured clinical interviews to verify ADHD diagnoses and systematically assess comorbid conditions, enabling statistical control for their independent contributions to functional outcomes.

Additionally, the ADHD symptom dimension analyses were constrained by the availability of Japanese normative data for the ADHD-RS-IV. The 95th percentile cutoffs used for presentation subtype classification have been established only for Grades 1–9 (elementary and junior high school), and no equivalent norms exist for Grades 10–12 (senior high school). Consequently, subtype classification was limited to 585 of the 797 ADHD group participants, with the 212 senior high school participants excluded from subtype analyses. Future research should prioritize the development of age-appropriate ADHD-RS cutoffs for Japanese senior high school students to enable comprehensive symptom dimension analysis across the full developmental range.

Relatedly, individual-level treatment data were not collected. Although population-level evidence indicates that ADHD medication use in Japan remains markedly low at 0.4% compared to 5.3% in the United States (38), with international comparisons across 13 countries confirming Japan among the lowest prescribing nations (61), these aggregate estimates cannot substitute for individual treatment histories. Pharmacological or psychosocial interventions may have differentially attenuated symptoms in a subset of the ADHD group, introducing unquantified variability in outcome measures that could not be controlled in the present analyses.

Additionally, although the survey confirmed that children in the ADHD group had received a formal diagnosis from a qualified healthcare professional, specific information about the diagnosing clinician’s medical specialty was not collected. In Japan, ADHD in children is typically diagnosed by child psychiatrists or pediatricians with developmental expertise (8, 40), but variation in diagnostic practices and expertise among physician specialties may exist. Future research should collect detailed information about the diagnostic context, including the clinician’s specialty, the setting in which the diagnosis was made, and whether standardized diagnostic instruments were employed.

Despite these limitations, this study contributes to understanding how ADHD impacts children’s emotional wellbeing and school functioning across developmental periods. The pronounced sex differences in mental health outcomes suggest the importance of sex-specific approaches to ADHD identification and intervention. Critically, the exclusive reliance on parent reports introduces a systematic informant bias that likely varies by child age. Informant discrepancies in the assessment of childhood psychopathology are well documented, with parents and children often providing divergent accounts of internalizing symptoms (78), and parent-adolescent disagreement on behavioral and emotional measures tends to increase during adolescence (79). In the present study, the reduction in effect sizes for internalizing symptoms at the senior high school stage may therefore reflect decreasing parental awareness rather than genuine symptom improvement—a pattern consistent with this tentative hidden-burden interpretation discussed above and with evidence that combined informant reports capture clinically significant impairment missed by single-informant assessment (68). Additionally, although sensitivity analyses controlling for household income were conducted, parental educational attainment—an important component of socioeconomic status—was not collected, limiting the comprehensiveness of socioeconomic adjustment. Future research should incorporate multi-informant assessment strategies integrating child self-reports, parent reports, and teacher ratings, alongside more comprehensive socioeconomic indicators, to provide a fuller account of ADHD-related impairments across development.

## Data Availability

The raw data supporting the conclusions of this article will be made available by the author, without undue reservation.
