# Supplementary material for: ADHD-related mental health and school impairments across educational stages: sex-specific patterns in Japanese youth
Source: Front Psychiatry. 2026 Oct 22;17:1736727. doi: 10.3389/fpsyt.2026.1736727 (PMC13736699; doi:10.3389/fpsyt.2026.1736727)
Supplement: Supplementary file 1 [file Table1.docx]

**Supplementary Table 1.**

Sample Size by Group

| **Sex** | **ADHD Diagnosis** | **School Level** | **N** |
| --- | --- | --- | --- |
| Girl | Yes | Elementary (Grades 1-6) | 76 |
| Girl | Yes | Junior high (Grades 7-9) | 75 |
| Girl | Yes | Senior high (Grades 10-12) | 65 |
| Girl | No | Elementary (Grades 1-6) | 124 |
| Girl | No | Junior high (Grades 7-9) | 128 |
| Girl | No | Senior high (Grades 10-12) | 136 |
| Boy | Yes | Elementary (Grades 1-6) | 218 |
| Boy | Yes | Junior high (Grades 7-9) | 216 |
| Boy | Yes | Senior high (Grades 10-12) | 147 |
| Boy | No | Elementary (Grades 1-6) | 141 |
| Boy | No | Junior high (Grades 7-9) | 139 |
| Boy | No | Senior high (Grades 10-12) | 129 |

**Supplementary Table 2.**

Three-Way ANOVA Results: Child Gender × ADHD Diagnosis × School Level

| **Dependent Variable** | **Effect** | **Df** | **Sum of Squares** | **F-value** | **p-value** |
| --- | --- | --- | --- | --- | --- |
| Support from Friends | childGender_factor | 1 | 0.40 | 0.51 | 0.474 |
| Support from Friends | adhdDiagnosis_factor | 1 | 23.03 | 29.39 | <0.001 |
| Support from Friends | schoolLevel | 2 | 0.29 | 0.19 | 0.829 |
| Support from Friends | childGender_factor:adhdDiagnosis_factor | 1 | 1.05 | 1.34 | 0.247 |
| Support from Friends | childGender_factor:schoolLevel | 2 | 1.51 | 0.96 | 0.383 |
| Support from Friends | adhdDiagnosis_factor:schoolLevel | 2 | 0.29 | 0.18 | 0.834 |
| Support from Friends | childGender_factor:adhdDiagnosis_factor:schoolLevel | 2 | 0.27 | 0.17 | 0.841 |
| Support from Friends | Residuals | 1,582 | 1239.61 | NA |  |
| Support from Teachers | childGender_factor | 1 | 8.93 | 10.57 | 0.001 |
| Support from Teachers | adhdDiagnosis_factor | 1 | 2.01 | 2.38 | 0.123 |
| Support from Teachers | schoolLevel | 2 | 0.85 | 0.50 | 0.605 |
| Support from Teachers | childGender_factor:adhdDiagnosis_factor | 1 | 3.12 | 3.70 | 0.055 |
| Support from Teachers | childGender_factor:schoolLevel | 2 | 4.38 | 2.60 | 0.075 |
| Support from Teachers | adhdDiagnosis_factor:schoolLevel | 2 | 2.79 | 1.65 | 0.192 |
| Support from Teachers | childGender_factor:adhdDiagnosis_factor:schoolLevel | 2 | 1.52 | 0.90 | 0.407 |
| Support from Teachers | Residuals | 1,582 | 1335.98 | NA |  |
| Academic Achievements | childGender_factor | 1 | 8.44 | 5.67 | 0.017 |
| Academic Achievements | adhdDiagnosis_factor | 1 | 67.59 | 45.37 | <0.001 |
| Academic Achievements | schoolLevel | 2 | 23.70 | 7.96 | <0.001 |
| Academic Achievements | childGender_factor:adhdDiagnosis_factor | 1 | 2.08 | 1.40 | 0.238 |
| Academic Achievements | childGender_factor:schoolLevel | 2 | 0.24 | 0.08 | 0.921 |
| Academic Achievements | adhdDiagnosis_factor:schoolLevel | 2 | 9.69 | 3.25 | 0.039 |
| Academic Achievements | childGender_factor:adhdDiagnosis_factor:schoolLevel | 2 | 1.22 | 0.41 | 0.664 |
| Academic Achievements | Residuals | 1,582 | 2356.50 | NA |  |
| Bullying Victimization | childGender_factor | 1 | 5.70 | 9.84 | 0.002 |
| Bullying Victimization | adhdDiagnosis_factor | 1 | 45.43 | 78.50 | <0.001 |
| Bullying Victimization | schoolLevel | 2 | 10.24 | 8.85 | <0.001 |
| Bullying Victimization | childGender_factor:adhdDiagnosis_factor | 1 | 5.09 | 8.79 | 0.003 |
| Bullying Victimization | childGender_factor:schoolLevel | 2 | 1.26 | 1.09 | 0.336 |
| Bullying Victimization | adhdDiagnosis_factor:schoolLevel | 2 | 4.93 | 4.26 | 0.014 |
| Bullying Victimization | childGender_factor:adhdDiagnosis_factor:schoolLevel | 2 | 0.60 | 0.52 | 0.595 |
| Bullying Victimization | Residuals | 1,582 | 915.64 | NA |  |
| Irritability (ARI) | childGender_factor | 1 | 53.52 | 7.23 | 0.007 |
| Irritability (ARI) | adhdDiagnosis_factor | 1 | 1513.99 | 204.62 | <0.001 |
| Irritability (ARI) | schoolLevel | 2 | 74.42 | 5.03 | 0.007 |
| Irritability (ARI) | childGender_factor:adhdDiagnosis_factor | 1 | 52.22 | 7.06 | 0.008 |
| Irritability (ARI) | childGender_factor:schoolLevel | 2 | 0.09 | 0.01 | 0.994 |
| Irritability (ARI) | adhdDiagnosis_factor:schoolLevel | 2 | 23.65 | 1.60 | 0.203 |
| Irritability (ARI) | childGender_factor:adhdDiagnosis_factor:schoolLevel | 2 | 2.76 | 0.19 | 0.830 |
| Irritability (ARI) | Residuals | 1,582 | 11705.04 | NA |  |
| Total Anxiety (SCAS) | childGender_factor | 1 | 15603.94 | 47.36 | <0.001 |
| Total Anxiety (SCAS) | adhdDiagnosis_factor | 1 | 60657.61 | 184.09 | <0.001 |
| Total Anxiety (SCAS) | schoolLevel | 2 | 6696.58 | 10.16 | <0.001 |
| Total Anxiety (SCAS) | childGender_factor:adhdDiagnosis_factor | 1 | 5994.58 | 18.19 | <0.001 |
| Total Anxiety (SCAS) | childGender_factor:schoolLevel | 2 | 1143.34 | 1.73 | 0.177 |
| Total Anxiety (SCAS) | adhdDiagnosis_factor:schoolLevel | 2 | 3306.19 | 5.02 | 0.007 |
| Total Anxiety (SCAS) | childGender_factor:adhdDiagnosis_factor:schoolLevel | 2 | 1602.23 | 2.43 | 0.088 |
| Total Anxiety (SCAS) | Residuals | 1,582 | 521256.31 | NA |  |
| Depression (SFMQ) | childGender_factor | 1 | 432.14 | 17.32 | <0.001 |
| Depression (SFMQ) | adhdDiagnosis_factor | 1 | 4522.83 | 181.30 | <0.001 |
| Depression (SFMQ) | schoolLevel | 2 | 277.40 | 5.56 | 0.004 |
| Depression (SFMQ) | childGender_factor:adhdDiagnosis_factor | 1 | 212.90 | 8.53 | 0.004 |
| Depression (SFMQ) | childGender_factor:schoolLevel | 2 | 75.36 | 1.51 | 0.221 |
| Depression (SFMQ) | adhdDiagnosis_factor:schoolLevel | 2 | 236.82 | 4.75 | 0.009 |
| Depression (SFMQ) | childGender_factor:adhdDiagnosis_factor:schoolLevel | 2 | 126.05 | 2.53 | 0.080 |
| Depression (SFMQ) | Residuals | 1,582 | 39466.67 | NA |  |

**Supplementary Table 3.**

Standardized Mean Differences (SMD): non-ADHD vs. ADHD by Sex and School Level

| **Dependent Variable** | **Sex** | **School Level** | **N (No)** | **M (No)** | **SD (No)** | **N (Yes)** | **M (Yes)** | **SD (Yes)** | **SMD** | **95% CI** |
| --- | --- | --- | --- | --- | --- | --- | --- | --- | --- | --- |
| Support from Friends | Girl | Elementary (Grades 1-6) | 124 | 3.58 | 0.76 | 76 | 3.14 | 1.04 | -0.50 | [-0.79, -0.21] |
| Support from Friends | Girl | Junior high (Grades 7-9) | 128 | 3.67 | 0.86 | 75 | 3.23 | 0.95 | -0.50 | [-0.79, -0.21] |
| Support from Friends | Girl | Senior high (Grades 10-12) | 136 | 3.56 | 0.74 | 65 | 3.22 | 0.99 | -0.41 | [-0.71, -0.12] |
| Support from Friends | Boy | Elementary (Grades 1-6) | 141 | 3.56 | 0.83 | 218 | 3.31 | 0.99 | -0.27 | [-0.48, -0.05] |
| Support from Friends | Boy | Junior high (Grades 7-9) | 139 | 3.54 | 0.78 | 216 | 3.16 | 0.96 | -0.42 | [-0.64, -0.21] |
| Support from Friends | Boy | Senior high (Grades 10-12) | 129 | 3.53 | 0.74 | 147 | 3.27 | 0.94 | -0.32 | [-0.55, -0.08] |
| Support from Teachers | Girl | Elementary (Grades 1-6) | 124 | 3.56 | 0.81 | 76 | 3.25 | 1.11 | -0.34 | [-0.62, -0.05] |
| Support from Teachers | Girl | Junior high (Grades 7-9) | 128 | 3.47 | 0.86 | 75 | 3.40 | 1.10 | -0.07 | [-0.36, 0.21] |
| Support from Teachers | Girl | Senior high (Grades 10-12) | 136 | 3.30 | 0.81 | 65 | 3.32 | 0.92 | 0.03 | [-0.27, 0.32] |
| Support from Teachers | Boy | Elementary (Grades 1-6) | 141 | 3.67 | 0.80 | 218 | 3.72 | 1.02 | 0.06 | [-0.16, 0.27] |
| Support from Teachers | Boy | Junior high (Grades 7-9) | 139 | 3.43 | 0.80 | 216 | 3.51 | 0.99 | 0.08 | [-0.13, 0.30] |
| Support from Teachers | Boy | Senior high (Grades 10-12) | 129 | 3.39 | 0.82 | 147 | 3.46 | 0.96 | 0.08 | [-0.15, 0.32] |
| Academic Achievements | Girl | Elementary (Grades 1-6) | 124 | 3.40 | 0.92 | 76 | 2.91 | 1.27 | -0.47 | [-0.75, -0.18] |
| Academic Achievements | Girl | Junior high (Grades 7-9) | 128 | 3.30 | 1.32 | 75 | 2.23 | 1.29 | -0.82 | [-1.11, -0.52] |
| Academic Achievements | Girl | Senior high (Grades 10-12) | 136 | 3.47 | 1.12 | 65 | 2.94 | 1.34 | -0.45 | [-0.74, -0.15] |
| Academic Achievements | Boy | Elementary (Grades 1-6) | 141 | 3.40 | 0.95 | 218 | 2.67 | 1.22 | -0.65 | [-0.86, -0.43] |
| Academic Achievements | Boy | Junior high (Grades 7-9) | 139 | 3.27 | 1.24 | 216 | 1.94 | 1.24 | -1.07 | [-1.29, -0.84] |
| Academic Achievements | Boy | Senior high (Grades 10-12) | 129 | 3.27 | 1.29 | 147 | 2.76 | 1.44 | -0.38 | [-0.61, -0.14] |
| Bullying Victimization | Girl | Elementary (Grades 1-6) | 124 | 1.13 | 0.51 | 76 | 1.96 | 1.10 | 1.06 | [0.75, 1.36] |
| Bullying Victimization | Girl | Junior high (Grades 7-9) | 128 | 1.09 | 0.33 | 75 | 1.48 | 0.95 | 0.62 | [0.33, 0.91] |
| Bullying Victimization | Girl | Senior high (Grades 10-12) | 136 | 1.04 | 0.29 | 65 | 1.54 | 1.08 | 0.75 | [0.45, 1.06] |
| Bullying Victimization | Boy | Elementary (Grades 1-6) | 141 | 1.24 | 0.65 | 218 | 1.78 | 1.14 | 0.55 | [0.33, 0.76] |
| Bullying Victimization | Boy | Junior high (Grades 7-9) | 139 | 1.13 | 0.55 | 216 | 1.40 | 0.88 | 0.35 | [0.13, 0.56] |
| Bullying Victimization | Boy | Senior high (Grades 10-12) | 129 | 1.04 | 0.26 | 147 | 1.23 | 0.67 | 0.37 | [0.13, 0.61] |
| Irritability (ARI) | Girl | Elementary (Grades 1-6) | 124 | 1.02 | 1.76 | 76 | 4.78 | 3.72 | 1.40 | [1.08, 1.72] |
| Irritability (ARI) | Girl | Junior high (Grades 7-9) | 128 | 0.77 | 1.63 | 75 | 4.20 | 3.50 | 1.38 | [1.06, 1.69] |
| Irritability (ARI) | Girl | Senior high (Grades 10-12) | 136 | 0.57 | 1.52 | 65 | 3.32 | 3.46 | 1.18 | [0.86, 1.50] |
| Irritability (ARI) | Boy | Elementary (Grades 1-6) | 141 | 1.45 | 2.01 | 218 | 4.19 | 3.43 | 0.92 | [0.70, 1.15] |
| Irritability (ARI) | Boy | Junior high (Grades 7-9) | 139 | 0.83 | 1.94 | 216 | 3.64 | 3.56 | 0.93 | [0.70, 1.15] |
| Irritability (ARI) | Boy | Senior high (Grades 10-12) | 129 | 0.64 | 1.38 | 147 | 2.71 | 3.06 | 0.85 | [0.60, 1.10] |
| Total Anxiety (SCAS) | Girl | Elementary (Grades 1-6) | 124 | 10.60 | 12.81 | 76 | 36.46 | 29.38 | 1.25 | [0.94, 1.56] |
| Total Anxiety (SCAS) | Girl | Junior high (Grades 7-9) | 128 | 8.21 | 11.84 | 75 | 31.01 | 24.46 | 1.30 | [0.98, 1.61] |
| Total Anxiety (SCAS) | Girl | Senior high (Grades 10-12) | 136 | 8.49 | 12.60 | 65 | 22.68 | 23.13 | 0.85 | [0.54, 1.15] |
| Total Anxiety (SCAS) | Boy | Elementary (Grades 1-6) | 141 | 10.02 | 15.12 | 218 | 26.22 | 25.48 | 0.74 | [0.52, 0.95] |
| Total Anxiety (SCAS) | Boy | Junior high (Grades 7-9) | 139 | 7.73 | 14.10 | 216 | 17.72 | 17.31 | 0.62 | [0.40, 0.84] |
| Total Anxiety (SCAS) | Boy | Senior high (Grades 10-12) | 129 | 4.37 | 6.86 | 147 | 16.16 | 18.09 | 0.84 | [0.59, 1.09] |
| Depression (SFMQ) | Girl | Elementary (Grades 1-6) | 124 | 1.60 | 3.15 | 76 | 9.11 | 7.43 | 1.44 | [1.12, 1.76] |
| Depression (SFMQ) | Girl | Junior high (Grades 7-9) | 128 | 2.23 | 4.26 | 75 | 7.47 | 6.68 | 0.99 | [0.69, 1.29] |
| Depression (SFMQ) | Girl | Senior high (Grades 10-12) | 136 | 1.91 | 3.44 | 65 | 6.32 | 5.97 | 1.00 | [0.69, 1.31] |
| Depression (SFMQ) | Boy | Elementary (Grades 1-6) | 141 | 2.33 | 3.92 | 218 | 6.63 | 6.15 | 0.80 | [0.58, 1.02] |
| Depression (SFMQ) | Boy | Junior high (Grades 7-9) | 139 | 1.88 | 3.63 | 216 | 6.63 | 5.66 | 0.96 | [0.73, 1.18] |
| Depression (SFMQ) | Boy | Senior high (Grades 10-12) | 129 | 1.20 | 3.11 | 147 | 4.63 | 5.43 | 0.76 | [0.52, 1.01] |
